# Characterization of the Early Inflammatory Infiltrate at the Feeding Site of Infected Sand Flies in Mice Protected from Vector-Transmitted *Leishmania major* by Exposure to Uninfected Bites

**DOI:** 10.1371/journal.pntd.0002781

**Published:** 2014-04-24

**Authors:** Clarissa Teixeira, Regis Gomes, Fabiano Oliveira, Claudio Meneses, Dana C. Gilmore, Dia-Eldin A. Elnaiem, Jesus G. Valenzuela, Shaden Kamhawi

**Affiliations:** 1 Vector Molecular Biology Section, Laboratory of Malaria and Vector Research, National Institute of Allergy and Infectious Diseases, National Institutes of Health, Rockville, Maryland, United States of America; 2 Centro de Pesquisas Gonçalo Moniz, Fiocruz, Bahia, Brazil; 3 Department of Zoology, University of Maryland Eastern Shore, Princess Anne, Maryland, United States of America; New York University, United States of America

## Abstract

**Background:**

Mice exposed to sand fly saliva are protected against vector-transmitted *Leishmania major*. Although protection has been related to IFN- γ producing T cells, the early inflammatory response orchestrating this outcome has not been defined.

**Methodology/Principal findings:**

Mice exposed to uninfected *P. duboscqi* bites and naïve mice were challenged with *L. major*-infected flies to characterize their early immune response at the bite site. Mostly, chemokine and cytokine transcript expression post-infected bites was amplified in exposed compared to naïve mice. In exposed mice, induced chemokines were mostly involved in leukocyte recruitment and T cell and NK cell activation; IL-4 was expressed at 6 h followed by IFN-γ and iNOS2 as well as IL-5 and IL-10 expression. In naïve animals, the transcript expression following *Leishmania*-infected sand fly bites was suppressed. Expression profiles translated to an earlier and significantly larger recruitment of leukocytes including neutrophils, macrophages, Gr^+^ monocytes, NK cells and CD4^+^ T cells to the bite site of exposed compared to naïve mice post-infected bites. Additionally, up to 48 hours post-infected bites the number of IFN-γ-producing CD4^+^T cells and NK cells arriving at the bite site was significantly higher in exposed compared to naïve mice. Thereafter, NK cells become cytolytic and persist at the bite site up to a week post-bite.

**Conclusion/Significance:**

The quiet environment induced by a *Leishmania*-infected sand fly bite in naïve mice was significantly altered in animals previously exposed to saliva of uninfected flies. We propose that the enhanced recruitment of Gr^+^ monocytes, NK cells and CD4 Th1 cells observed at the bite site of exposed mice creates an inhospitable environment that counters the establishment of *L. major* infection.

## Author Summary

Sand flies transmit *Leishmania* parasites during bloodfeeding. Salivary molecules are deposited alongside parasites and can reshape the host's immune response to infection. Exposure to uninfected sand fly bites or immunization with salivary molecules protects the host against *Leishmania* infection. Here we show that mice exposed to bites of uninfected *Phlebotomus duboscqi* sand flies are protected against *P. duboscqi*-transmitted *L. major* and characterize the formerly unknown early cellular infiltrate at the bite site following *L.major* vector-transmission. The kinetics and nature of the inflammatory response at the bite site of exposed mice were notably different from those of naïve mice showing an amplified expression of cytokines and chemokines after parasite transmission. The transcripts reflected a faster and more robust infiltrate of immune cells to the bite site of exposed mice composed of neutrophils, macrophages, monocytes, NK cells and CD4+ T cells. In addition, there was an increased influx of activated IFN-γ producing CD4+ T cells and Granzyme B-producing mature NK cells in exposed animals. These findings suggest that the observed robust and persistent proinflammatory response in exposed animals restrict parasite multiplication.

## Introduction

Early studies demonstrated that mice exposed to saliva of the vector sand fly *Phlebotomus papatasi* develop immunity to its salivary proteins that confers powerful protection against *Leishmania major* [1]–[3]. These initial observations were validated by the identification of distinct salivary proteins from various vector species that conferred protection from *Leishmania* infection in immunized animals [4]–[9]. Further characterization of the adaptive immune response to saliva demonstrated that it is cell-mediated and dependent on IFN-γ-producing Th1 CD4 cells [5], [6], [10], [11].

In naïve animals, *Leishmania* parasites as well as sand fly saliva have each been associated with suppression of the initial proinflammatory immune response thereby promoting parasite survival [7], [11]–[18]. Co-injection of saliva or its components together with *Leishmania* parasites was shown to exacerbate cutaneous leishmaniasis (CL) infections producing larger lesions and a higher parasite burden. Enhancement of *Leishmania* infections by saliva was attributed to the immunomodulatory properties of salivary proteins that act early during infection to promote downregulation of dendritic cells and macrophage function and the production of anti-inflammatory cytokines that favor parasite establishment [19]–[23]. *Leishmania* parasites also orchestrate their own suppression and deactivation of the function of immune cells including macrophages [24]–[30] and NK cells [31], [32] recruited to the site of infection promoting their survival.

Additionally, in the context of vector-transmission, the parasite-derived promastigote secretory gel (PSG) was also shown to modulate the bite site to the advantage of the parasites. PSG shed by the *Leishmania* in the sand fly midgut is regurgitated by the fly during an infective bite and was shown to exacerbate *Leishmania* infection by acting on host macrophages to promote their alternate activation and parasite survival [33].

In any natural transmission event, an infected sand fly bites the host co-injecting saliva, PSG and parasites into the wound. We postulate that during vector-transmission of *Leishmania* to animals previously exposed to saliva or immunized with a protective salivary molecule, a rapid influx of immune cells is recruited to the bite site by the deposited saliva creating a proinflammatory environment that overcomes the suppressive nature of saliva, PSG and *Leishmania*. Such an inflamed bite site in exposed animals is likely to adversely affect the co-deposited parasites controlling their growth at an early stage and permitting a slower, damage-limited, development of *Leishmania*-specific immunity and conferring long-term protection from leishmaniasis [5], [6], [10], [11]. Here, we investigate the kinetics and phenotype of the cellular infiltrate arriving at the site of an infective bite in saliva-exposed mice compared to naïve animals to elucidate the significance of the early inflammatory response in saliva-mediated protection from leishmaniasis.

## Methods

### Ethics statement

The Animal Care and Use Committee at the National Institute of Allergy and Infectious Diseases adheres to the U. S. Government Principles for the Utilization and Care of Vertebrate Animals Used in Testing, Research, and Training and maintains animals in accordance with the PHS Policy on Humane Care and Use of Laboratory Animals, the

Guide for the Care and Use of Laboratory Animals, and the Animal Welfare Act and Animal Welfare Regulations user guidelines and has reviewed and approved all experimental procedures involving animal use for this study under animal protocol LMVR 4E.

### Mice

C57BL/6 female mice, 4–6 weeks old (Charles River Laboratories) were maintained under pathogen free conditions.

### Parasites and sand fly infection

*Leishmania major* clone V1 (MHOM/IL/80/Friedlin) amastigotes were used for sand fly infection. Amastigotes were washed with PBS, counted and mixed with mouse blood. Before mixing, mice serum was heated for one hour at 56°C. Blood containing 3×10^6^ *L. major*/ml were used to artificially feed sand flies using a glass chamber covered with a chicken skin membrane as previously described [2]. Blood-fed sand flies were separated 24 hours after infection and kept contained in secure paper cups in an incubator at 25°C and 75% relative humidity. Flies were offered 30% sucrose in soaked cotton balls. Before transmission sand flies were dissected to examine the quality of the infection and quantity of metacyclic promastigotes as previously described [34]. Sand flies were used for transmission experiments on days 13 to 14 after infection.

### Exposure to sand flies

*Phlebotomus duboscqi* (Mali strain) sand flies were reared at the Laboratory of Malaria and Vector Research, NIAID. Exposure of mice to uninfected flies was carried out on a weekly basis for a total of three exposures. *P. duboscqi* sand flies used for these experiments were emergent females (5–7 day old) left without sugar overnight. Mice were anesthetized intraperitoneally with ketamine (Phoenix Pharmaceuticals, St. Joseph, MO) according to the weight of the animal. Ten sand flies were placed in plastic vials covered at one end with a 0.25 mm nylon mesh and left to feed on the right ear in the dark. The ear of anesthetized mice was pressed flat against the mesh surface of the vial containing the flies using custom-designed clamps. Sand flies were allowed to feed for up to 30 minutes and were then examined for blood to assess exposure success. For challenge, 10 *L. major*-infected sand flies were placed in individual vials and applied to the left ear of mice two weeks after the last exposure to uninfected flies as described above. Infected flies were applied to the contralateral ear of exposed mice to demonstrate that exposure to uninfected flies generates systemic immunity. Infected sand flies were allowed to feed for two hours in the dark after which they were examined for blood to assess the success of transmission

### Lesion size and limiting dilution assay

For measurement of *Leishmania* lesions the largest diameter was recorded on a weekly basis using a Digimatic caliper (Mitutoyo). Parasite quantification was performed using a limiting dilution assay as previously described [1], [35]. Briefly, total ear tissue homogenates were serially diluted (1∶5) in 96-well flat bottom microtiter plates containing 50 µl biphasic medium prepared using NNN medium with 10% of defibrinated rabbit blood overlaid with 200 µl Schneider's (Gibco, NY) supplemented with 20% heat inactivated fetal bovine serum, 2 mM L-glutamine, 100 U/ml penicillin and 100 µl/ml streptomycin. The number of viable *Leishmania* in each ear tissue was determined from the highest dilution at which *Leishmania* promastigotes could be grown after 7 days of incubation at 26°C.

### RNA extraction and array analysis

The expression profile of cytokines, chemokines, and related inflammatory genes was generated using the mouse inflammatory cytokines and receptor Oligo GEArray (OMM-011; Superarray). This array contains 112 genes representing cytokines, receptors and housekeeping genes. Six and 12 hours after exposure to uninfected sand flies or after challenge with *L. major*-infected sand flies, total RNA was isolated from the left ear of each mouse using the RNeasy Mini Kit (Qiagen) according to the manufacturer's instructions. RNA (6 µg) from a pool of five ears was amplified and labeled with biotin 16-UTP (Roche Diagnostics) using the SuperArray TrueLabeling-RT Enzyme kit (Superarray). The resulting biotinylated cRNA was hybridized overnight to the Oligo GEArray membrane. After washing and blocking the array membranes, alkaline phosphatase-conjugated streptavidin was added to the membrane followed by the CDP-Star substrate. A chemiluminescent signal was acquired using the Image Station 2000 MM (Kodak). The data was analyzed using the GEArray Expression Analysis Suite (Superarray). Analysis parameters were set to local background correction and normalized to a set of housekeeping genes included in each membrane. Results were expressed as fold increase in the intensity of the captured signal over the control group (ear tissue not exposed to bites). Only genes showing at least a four-fold or higher change in expression in at least three out of four independent experiments were considered.

### Real time PCR

The genes that showed a four-fold or higher change in expression over the control group (ear tissue not exposed to bites) using the GEArray were validated by Real time PCR. Total RNA from individual mice ears was used for the synthesis of cDNA (Superscript III, Invitrogen) following the manufacturer's instructions. The cDNA was amplified with the 480 Master SYBR Green I mix (Roche Diagnostics) and gene specific primer sets for IFN-γ, IL-4, CXCL13 and CCL25 using the LightCycler 480 (Roche Diagnostics). A standard curve for each set of primers was generated as recommended by the manufacturer. The expression levels of the genes of interest were normalized to endogenous 18S RNA levels. The results are expressed in fold change over gene expression in the control group.

### Preparation of BMDC

Bone Marrow Dendritic Cells (BMDCs) were obtained from the femurs of mice and cultured in RPMI medium enriched with 10% heat-inactivated fetal bovine serum (HyClone), 100 U/ml penicillin, 100 µg/ml streptomycin, 2 mM L-glutamine, 40 mM Hepes, 5×10^−5^ M 2-mercaptoethanol, plus 20 ng/ml GM-CSF (Prepotech). The medium was replaced by fresh complete RPMI containing 10 ng/ml of GM-CSF on day 3.

### Cell suspensions and flow cytometry staining

Cells were recovered from the ear dermis as described previously (Belkaid et al, 1998; Peters et al, 2008). For characterization of leukocyte populations the cells were washed, incubated for 30 min at 4°C with anti-CD16/CD32 (BD Fc block, 2.4G2; BD Pharmingen), and stained *ex vivo* with a combination of surface markers including PerCP-labeled anti-CD4 (RM4-5) or PE -labeled anti-CD4 (GK1.5), FITC-labeled anti-Ly6G (RB6-8C5), APC-labeled anti-TCR-β (H57-597), PerCP-Cy5.5 labeled anti-NK1.1 (PK136), PE- or PerCP-labeled anti-CD11b (M1/70), APC-labeled anti-F4/80 (BM8), for 30 minutes at 4°C. For intracellular stains, cells (2×10^6^) were cultured with 2.5×10^5^ Bone Marrow Dendritic Cells (BMDCs) in one mL of RPMI 1640 containing 10% FBS, L-glutamine and penincilin/streptomycin in flat-bottom 48-well plates at 37°C and 5% CO_2_ for 18 h with the addition of 20 ng of PMA and 500 ng of ionomycin and 1 ul of Brefeldin A (BD Golgi Plug; BD Pharmingen) during the last 4 h of culture. The cells were then washed with PBS and blocked with anti-CD16/CD32 (BD Fc block, 2.4G2; BD Pharmingen) for 30 minutes at 4°C. Cells were stained with a combination of PerCP-labeled anti-CD4 (RM4-5) or PE-labeled anti-CD4 (GK1.5), APC-labeled anti-TCR-β (H57-597), PerCP-Cy5.5 labeled anti-NK1.1 (PK136), FITC or PerCP-Cy5.5-labeled anti-CD11b (M1/70) and PE-labeled anti-CD27 (LG.3A10) or 30 minutes at 4°C, washed twice, fixed and permeabilized with Cytofix/Cytoperm Plus (BD Pharmingen) and stained with FITC-labeled anti-IFN-γ (XMG 1.2) and/or APC-labeled anti-Granzyme B (GRB05, Invitrogen). A minimum of 100,000 cells was acquired using a FACSCalibur flow cytometer (BD Biosciences). The data were analyzed using the Flow Jo software.

### Statistical analysis

Graphs and statistical significance were prepared and analyzed using GraphPad Prism Software 5.0 (GraphPad, San Diego, CA). Data from parasite numbers were log transformed before conducting statistical tests. The unpaired *t* test with Welch's correction or one-way analysis of variance followed by the Tukey-Kramer post-test was used to evaluate statistical significance among groups. A *p* value<0.05 was considered statistically significant.

## Results

### Exposure to bites of uninfected *Phlebotomus duboscqi* sand flies protects against vector-transmission with *Leishmania major*

**Figure 1:**
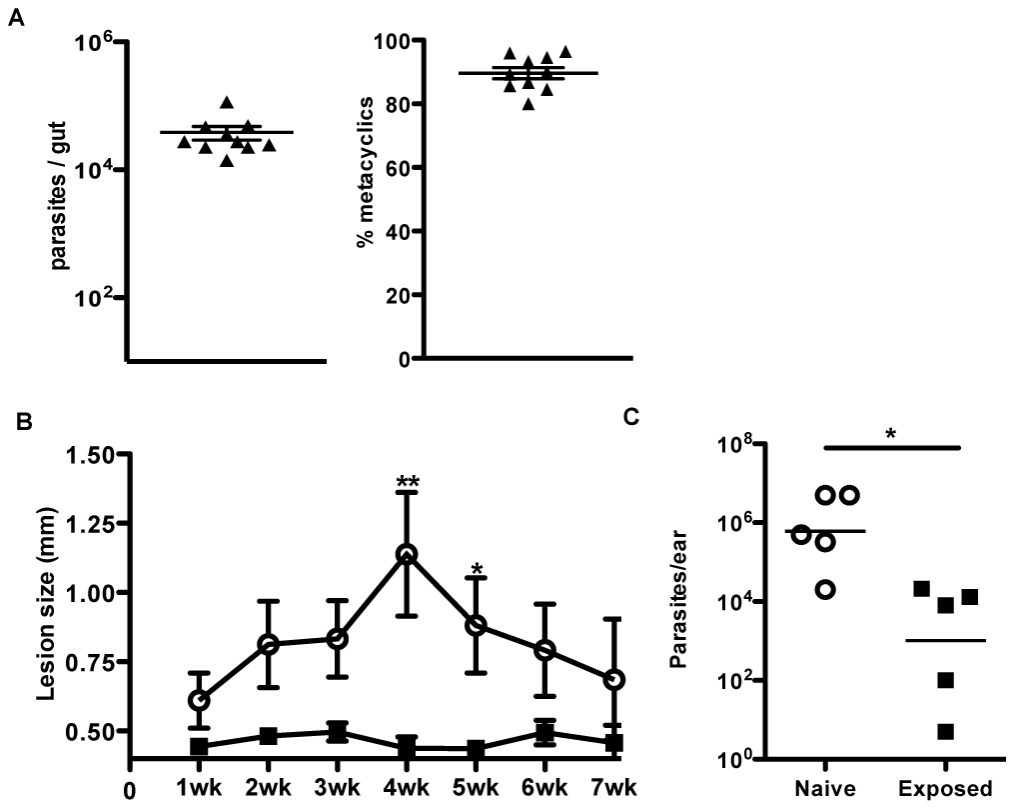
Mice exposed to uninfected *P. duboscqi* bites are protected against vector-transmitted *L. major*. (**A**) Representative parasite load and percent metacyclics in the gut of infected *P. duboscqi* sand flies the day of transmission. The mean ± SEM are shown. (**B–C**) Mice exposed to uninfected bites in the right ear (▪) or are naïve (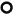) were challenged with 10 *L. major*-infected *P. duboscqi* sand flies in the left ear two weeks after the last exposure. (**B**) Weekly measurement of ear lesions after transmission. The mean ± SEM for 10 mice per group are shown. (**C**) The number of parasites per ear for five mice at four weeks post-transmission determined by limiting dilution assay. The bar represents the mean parasite burden per ear. Data are representative of three independent experiments. *p<0.02; **p = 0.008.

Mice were exposed to bites of uninfected *P. duboscqi* sand flies three times at two-week intervals and were challenged alongside naïve mice with ten *L. major*-infected sand flies two weeks after the final exposure. A mean parasite load of 3.8×10^4^ and a mean percent metacyclics of 89% are representative of the infection status of sand flies used in transmission (Fig. 1A). Saliva-exposed mice controlled the infection while naïve mice displayed an increasing lesion size that peaked four weeks post-infection (Fig. 1B). Additionally, there was a three log reduction (p = 0.016) in the number of parasites recovered from the ear of exposed compared to naïve mice (Fig. 1C).

### Expression of inflammatory cytokines at the bite site six to 12 hours post vector-transmission of *L. major*

**Figure 2:**
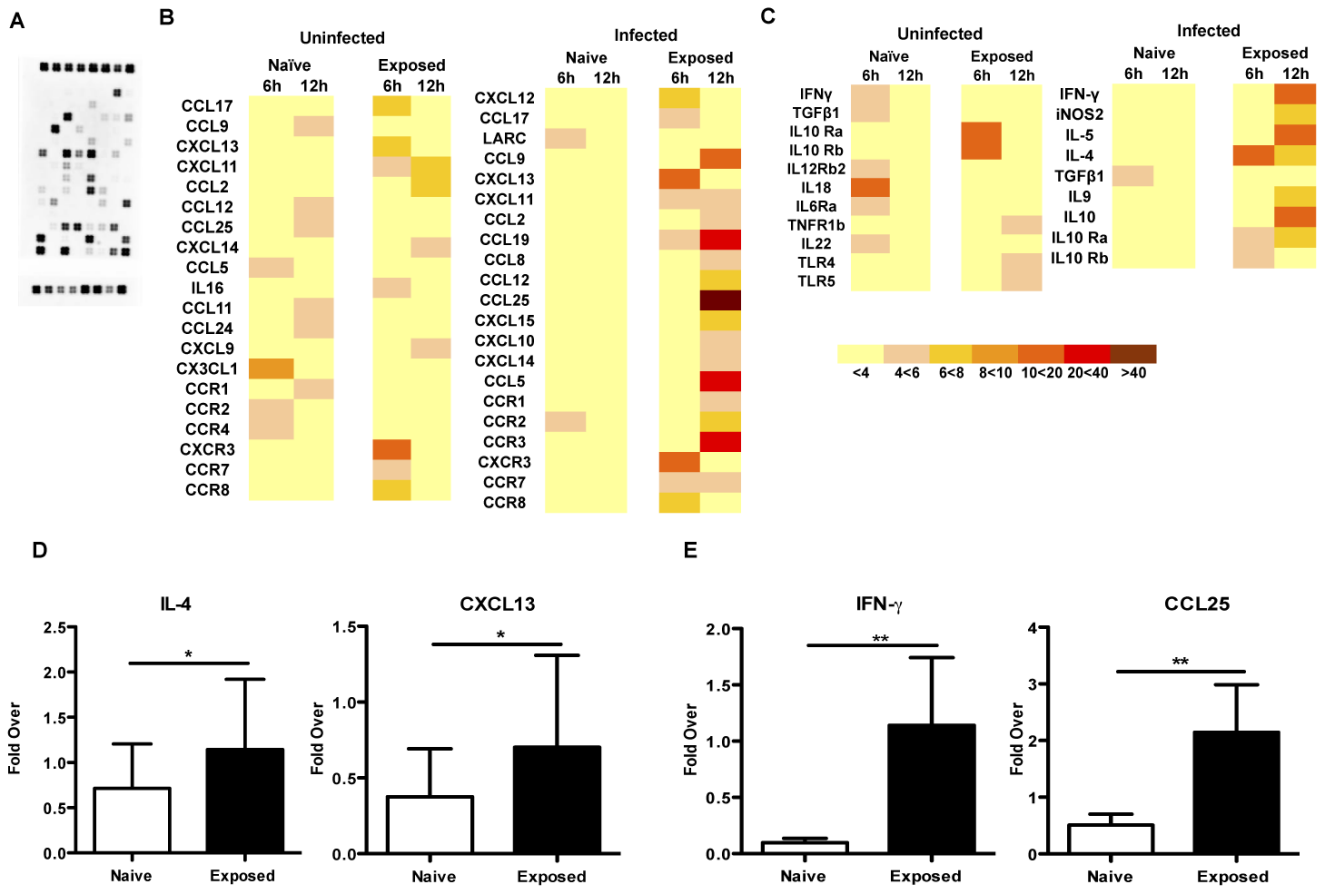
The expression of inflammatory transcripts in naïve and sand fly-exposed mice following vector-transmitted *L. major*. Mice exposed to uninfected bites in the right ear or naïve mice were challenged with 10 *L. major*-infected *P. duboscqi* sand flies (infected) or 10 uninfected sandflies (uninfected) in the left ear. Six and 12 hours following sand fly transmission, RNA was extracted from the challenged ear of 5 naïve or exposed mice and pooled. (**A**) Picture of a representative array after RNA hybridization and chemiluminescent detection. Spots were identified using the gene array map and the expressed transcripts were normalized to housekeeping genes. Results are expressed as fold increase in the intensity of the captured signal over the control group. The values are depicted as heat maps using a color code to indicate relative levels of chemokine (**B**) and cytokine (**C**) expression. Validation of mRNA expression by quantitative Real Time PCR is shown for IL-4 and CXCL13 at 6 h (**D**) and IFN-γ and CCL25 at 12 h (**E**) after transmission. Only genes showing at least a four-fold or higher change in expression in at least three out of four independent experiments are shown. Values represent relative transcript expression for 10 mice per group. *p<0.05, **p≤0.0001.

The expression of inflammatory cytokines in the ear tissue of saliva-exposed and naïve mice was determined at 6 h and 12 h after exposure to uninfected sand flies or after challenge with *L. major*-infected sand flies using the Oligo GEArray Mouse Inflammatory Cytokines and Receptors Microarray that targets key genes involved in inflammation (Fig. 2A). The genes with a reproducible four-fold or higher change in signal intensity compared to unbitten ear tissue are represented in a heat map (Fig. 2B, 2C). Transcript expression was distinct in naïve compared to exposed mice following uninfected bites. Following infected vector bites, the expression profile of inflammatory genes in sand fly-exposed mice was amplified and displayed a dramatic difference compared to naïve mice (Fig. 2B, 2C). Interestingly, the presence of *Leishmania* parasites augmented gene expression in exposed mice but had a suppressive effect on naive animals (Fig. 2B, 2C). In exposed mice, the induced chemokine receptors and ligands were mostly involved in leukocyte recruitment and T and NK cell activation. Additionally, some genes related to allergic responses were also induced. Certain chemokines were induced by both uninfected and infected sand fly bites including CCL17, CXCL11, CXCL13, CXCR3, CCR7 and CCR8 at 6 h, and CXCL11, CCL2 and CXCL14 at 12 h post-transmission. Of those, CXCR3 and CXCL13 expression were the most prominent. CXCR3 is preferentially expressed on activated Th1 and NK cells and its ligand interactions promote polarization towards a Th1 effector immune response while CXCL13 selectively attracts B cells. Nevertheless, the presence of *Leishmania* parasites modulated the inflammatory response in exposed mice, particularly at 12 h after transmission. The inflammatory response of sand fly-exposed mice to infected bites intensified with a strong expression of CCL9, CCL19 and CCL25, chemokines known to recruit lymphocytes, monocytes and dendritic cells (Fig. 2B). CCL5, a chemokine that recruits leukocytes and can activate NK cells in the presence of IFN-γ or IL-2 and CCR3, a receptor highly expressed in eosinophils and basophils but is also detected in T cells, were also highly induced (Fig. 2B). A similar pattern was observed for cytokine expression. Compared to uninfected bites, there was a generalized suppressive effect on cytokine expression in naïve animals following infected bites while exposed mice showed an augmented response (Fig. 2C). Sand fly-exposed animals expressed IL-4 at 6 h after transmission and a more pronounced IFN-γ and iNOS2 as well as IL-5 and IL-10 at 12 h (Fig. 2C). In contrast, the naïve group again displayed an overall quiet response with only a moderate expression of TGF-β at 6 h (Fig. 2C). The array expression profiles were validated by real-time PCR for several genes (Fig. 2D, 2E).

### Characterization of the early inflammatory response at the bite site after vector-transmission of *L. major*

**Figure 3:**
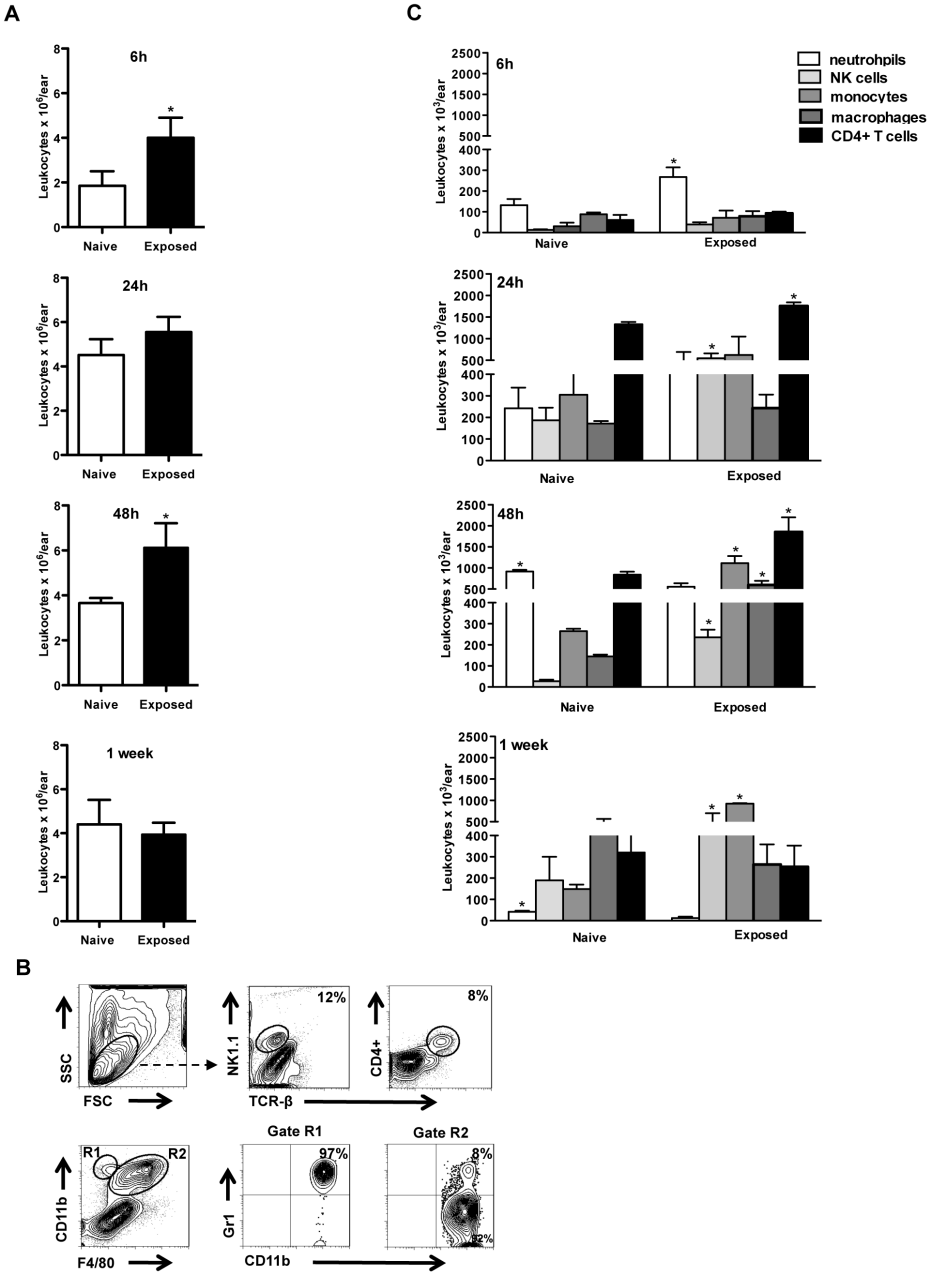
Characterization of the early leukocyte infiltrate following vector-transmission of *L. major*. Ear cells were recovered from naïve and exposed mice at 6 h, 24 h, 48 h and one week after transmission. At the indicated times after challenge, cells were stained *ex vivo* and analyzed by FACS for surface expression of TCR-β, CD4, NK1.1, F4/80, CD11b, and Ly-6G to identify specific leukocyte populations. (**A**) Absolute number of leukocytes per ear; (**B**) The gating strategy used to identify cells (NK1.1^pos^TCR-β^neg^); CD4^+^ T cells (TCR-β^pos^CD4^pos^), neutrophils (F4/80^neg^ CD11b^high^Ly6G^high^), Gr1^+^ inflammatory monocytes (F4/80^pos^CD11b^high^Ly6G^high^) and macrophages (F4/80^high^Ly6G^neg^CD11b^high^); (**C**) Absolute number of neutrophils, NK cells, Gr1^+^ monocytes, macrophages and CD4^+^ T cells per ear. The numbers shown represent mean ± SEM from 3 independent experiments. Five mice were used per group per experiment. *p<0.05.

Focusing on infected bites, we investigated the kinetics and phenotype of the cells recruited to the bite site at distinct time points up to one week after transmission. The augmented gene expression observed at the bite site of saliva-exposed mice was reflected in the local inflammatory response following transmission. Generally, the number of leukocytes increased with time in both naïve and exposed mice. However, exposed animals sustained a higher number of leukocytes compared to naïve mice at 6 h, 24 h and 48 h post-transmission that was significant (*p*<0.05) at 6 h and 48 h (Fig. 3A). The peak of cell recruitment in exposed animals was observed 48 h post-transmission (Fig. 3A). By one week post-transmission, the number of leukocytes present at the bite site was similar in both groups. Of note, cellular infiltration at the bite site was considerably faster in the exposed group with a mean of 3.2×10^6^ recruited cells per ear at 6 h post-transmission compared to only 1.5×10^6^ cells per ear in naïve mice (Fig. 3A).

We further characterized the major cell types comprising the leukocyte infiltrate after infected sand fly bites (Fig. 3B). Compared to naïve animals, exposed mice recruited a significantly higher number of neutrophils to the skin 6 h post-transmission (Fig. 3C). By 48 h all cell populations analyzed apart from neutrophils (NK cells, CD4+ T cells, inflammatory Gr1+ monocytes and macrophages) were present in significantly higher numbers (*p*<0.05) at the bite site of exposed compared to naïve mice (Fig. 3C). At this timepoint, the number of recruited neutrophils was significantly higher in naïve compared to exposed animals. One week post-infected bites, the number of neutrophils had subsided in both naïve and exposed mice (Fig. 3C). In contrast, NK cells and Gr1+ monocytes persisted in the bite site of exposed mice in a pronounced manner that was significantly higher (*p*<0.05) compared to naive mice a week post-infected bites (Fig. 3C).

### Activation state of CD4+ T cells and NK cells at the bite site following vector-transmission of *L. major*

**Figure 4:**
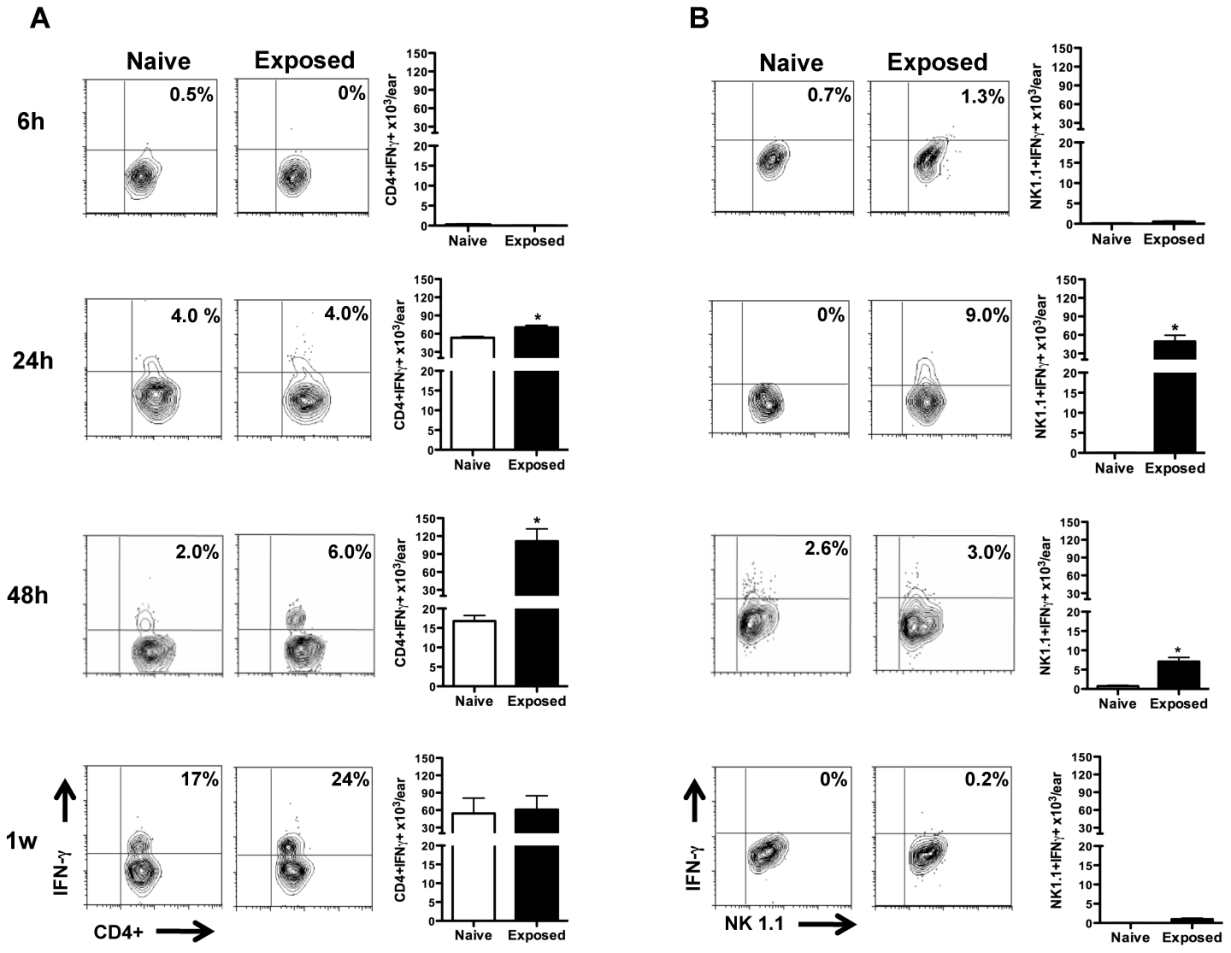
IFN- γ expression by T cells and NK cells following infected sand fly bites. Ear cells were recovered from naïve and exposed mice at 6 h, 24 h, 48 h and one week after transmission and cultured for 16 h. Brefeldin, PMA and ionomycin were added during the last four hours. The percent and absolute number of CD4+ T cells (**A**) or NK cells (**B**) expressing IFN-γ. Percentages shown are representative of three independent experiments; absolute numbers shown represent the mean ± SEM from three independent experiments. Five mice were used per group per experiment. *p<0.05.

We were interested to further explore the activation state of CD4+ T cells and NK cells recruited to the bite site. The percent of IFN-γ-producing CD4+ T cells increased with time for both exposed and naïve mice (Fig. 4A). At 24 h and 48 h post-transmission, the absolute number of IFN-γ-producing CD4+ T cells was significantly higher in exposed (70×10^3^ and 92×10^3^, respectively) compared to naïve animals (51×10^3^ and 16.7×10^3^ respectively) reflecting a more rapid and intense recruitment of these cells in exposed mice (Fig. 4A). One week post-transmission, the naïve mice recruited a comparable number of IFN-γ-producing CD4+ T cells to the bite site (Fig. 4A).

**Figure 5:**
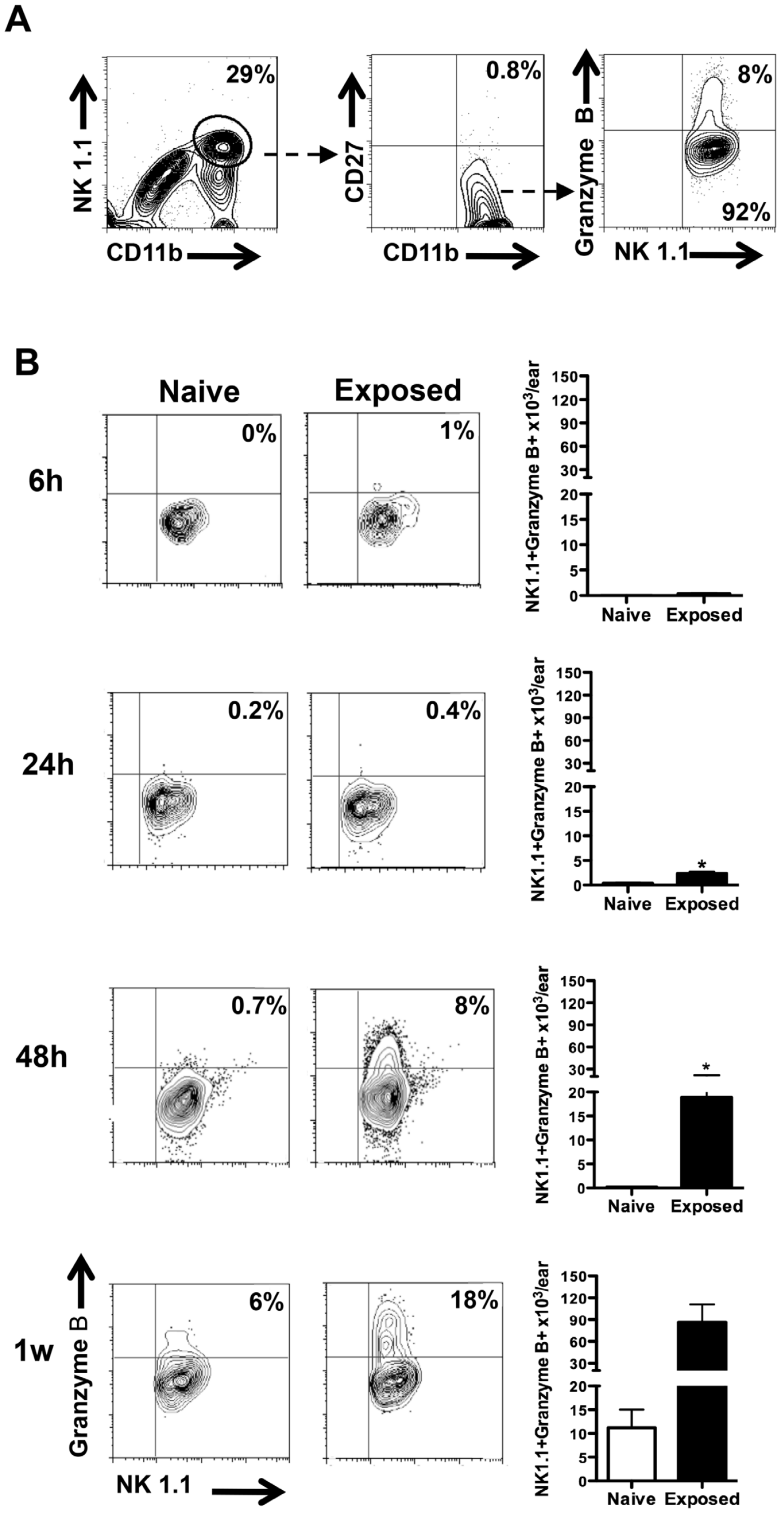
Mature NK cells expressing Granzyme B persist in exposed mice following infected sand fly bites. Ear cells were recovered from naïve and exposed mice at 6 h, 24 h, 48 h and one week after transmission and cultured for 16 h. Brefeldin, PMA and ionomycin were added during the last four hours. (**A**) The gating strategy used to identify NK cells. (**B**) The percent and absolute number of NK cells producing granzyme B in the NK1.1^pos^CD11b^pos^CD27^neg^ population. Percentages shown are representative of three independent experiments; absolute numbers shown represent the mean ± SEM from 3 independent experiments. Five mice were used per group per experiment. *p<0.05.

Similarly, 24 h and 48 h post-transmission, the absolute number of IFN-γ-producing NK cells was significantly higher in exposed (34.8×10^3^ and 6.5×10^3^, respectively) compared to naïve mice (zero and 0.7×10^3^, respectively) (Fig. 4B). Surprisingly, despite a significant increase in the number of NK cells recruited to the bite site of exposed mice at the one-week timepoint (Fig. 3B), very few were IFN-γ-producing cells (Fig. 4B). Gating on mature CD11b^+^CD27^−^Granzyme^+^ NK cells (Fig. 4C), 8% (16×10^3^) of NK cells present at the bite site of exposed mice displayed a cytolytic phenotype 48 h post-transmission (Fig. 5). This number increased to 18% (67×10^3^) a week post-transmission (Fig. 5). Of note, compared to naïve mice, the number of cytolytic NK cells observed at the bite site of exposed mice was 50- and 6-fold higher 48 h and one week post-transmission respectively (Fig. 5).

## Discussion

Transmission of *Leishmania* parasites by an infected sand fly bite is a complex event. During blood feeding, a cocktail of parasites, saliva and PSG, each with distinct immunomodulatory properties, is deposited into the host's skin. These components together with tissue damage caused by probing trigger a potent inflammatory response to an infected bite. The development of adaptive immunity to *Leishmania* parasites is dependent on the nature of the inflammatory response triggered during the first hours after infection where the local environment dictates what happens downstream of the immune response [36]. We have previously demonstrated that adaptive immunity to sand fly saliva or certain of its immunogenic proteins confers protection against vector-transmitted *L. major* [2], [10]. Similarly, the initial inflammatory response to *L. major* following needle injection of the parasites has been well described [37]–[41]. However, the immune response to needle-injected *Leishmania* parasites differs significantly from that observed following vector-transmission [2], [10], [14], [42]. Additionally, only one study compared the acute immune response to infected vector bites in naïve mice compared to animals protected by exposure to *P. papatasi* saliva, a sand fly vector of CL [2]. The authors reported a 9- and 15-fold higher IFN-γ- and IL-12-producing cells, respectively, at the bite site in exposed compared to naïve mice six hours post-transmission [2]. In exposed mice, this initial response was followed by a strong delayed–type hypersensitivity response that was associated to the observed protection from CL [2].

Here, we first established that exposure to bites of uninfected *P. duboscqi* sand flies, another important vector of CL, confers protection against vector-transmitted *L. major*. Next, we determined the nature of the inflammatory response at the bite site of exposed mice compared to naïve animals following uninfected and *Leishmania*-infected sand fly bites. Interestingly, the inflammatory response to uninfected and infected bites in naive compared to exposed mice was different. In both cases, the inflammatory response was weaker in naïve mice and was further suppressed by *Leishmania* parasites. In contrast, the number and level of chemokines and cytokines expressed in exposed mice was augmented as early as 6 h and 12 h following vector-transmission of *Leishmania* parasites suggesting that immunity to saliva overcomes immune suppression by the parasites that are known to down-modulate the immune response [18], [43]. The hyper-induction of cytokines and chemokines in the exposed mice translates to a more intense leukocyte infiltration compared to the one observed for naïve mice 24–48 h post infected bites. Our findings are distinct from those of Carregaro et al. [11] that noted a decrease in the majority of cell types including neutrophils, macrophages and CD4 T cells recruited to ears of BALB/c mice exposed to *Lutzomyia longipalpis* saliva compared to naive mice 24 h post-needle challenge with saliva. The observed differences may be attributable to several parameters that varied between the two studies including the mice strains, sand fly vector species and mode of exposures/challenge used. Of note, the magnitude of the cellular response observed in both naïve and exposed mice in the present study is stronger than reported by studies using needle-injected parasites [11], [15] or a lower number of sand flies [14] and emphasizes the potency of vector-transmission.

In the present study, the infiltration of leukocytes to the skin at the bite site was amplified in exposed compared to naïve mice, beginning with neutrophils at 6 h post-transmission and expanding to include macrophages, monocytes, NK cells and CD4 lymphocytes at 24–48 h. Interestingly, the pattern of cell recruitment for neutrophils and NK cells may be indicative of the occurrence of two waves of cell recruitment that possibly contribute to the persistence of cells at the bite site in exposed mice. Recruitment of neutrophils to the bite site was significantly larger in exposed mice at 6 h and was maintained up to 48 h. In contrast, neutrophils were only higher in naïve mice at 48 h after infected bites. The response subsided in both the naïve and exposed groups by one week post-transmission. This differs from recent findings that describe the persistence of neutrophils up to 1–4 weeks in large numbers at the bite site as a feature of vector-transmission to naïve mice where they provide safe passage of parasites into macrophages [14], [42]. Peters et al. [14] demonstrated that neutrophils rescue *L. major* parasites, promoting a silent entry of parasites into macrophages thereby enhancing their survival. Similarly, neutrophils were quickly recruited to the injection site of *L. infantum* and contained the majority of intracellular parasites up to 24 h after infection [36]. Taking into consideration that these studies were conducted only in naïve animals, it is clear from our transcript expression data that the suppression exerted by *Leishmania* parasites in naïve animals is overcome in exposed mice. We hypothesize that due to an intensified proinflammatory environment at the bite site of exposed animals, the activation state and nature of the interaction between macrophages and neutrophils may be altered to the detriment of the parasites contributing instead to disease control. Certainly, the number of recruited macrophages as well as Gr1+ inflammatory monocytes was significantly higher in exposed compared to naïve mice at 48 h post-transmission. A recent study showed that inflammatory monocytes are rapidly recruited to *L. major* lesions and contribute to killing of the parasites [44]. These inflammatory monocytes express the chemokine receptor CCR2 and are recruited by locally induced CCL2 from activated platelets [44]. Here, compared to naïve mice, exposed animals expressed elevated levels of both CCL2 and CCR2 12 hours post-transmission that correlate to the increased migration of Gr1+ monocytes to the site of bite at later timepoints where may contribute to early parasite control.

The proinflammatory nature of the bite site in exposed mice corresponds to a rapid and sustained recruitment of NK and CD4 T cells to the ears of exposed mice. The phenotype of the CD4+ T cells and NK cells was dramatically different in exposed and naïve groups; both cell types produced significantly higher levels of IFN-γ gamma in exposed compared to naïve mice as early as 24 h (NK cells) and 48 h (CD4+ T cells) post-infected bite. This upholds previous findings where protection from *Leishmania* observed in animals exposed to uninfected sand fly bites or following immunization with a single salivary molecule consistently correlated with the development of a DTH response and IFN-γ production around 48 h post-infection [10]. NK cells in exposed mice further develop into a CD27−/granzyme+ cytotoxic phenotype at 48 h and persist at the bite site up to a week post-infected bites. Many studies have implicated activated NK cells in protection from *Leishmania* [32], [45]–[48], however, their significance following vector-transmission of *L. major* needs to be further elucidated.

Taken together, the results obtained here demonstrate that exposure to *P. duboscqi* saliva generates a strong inflammatory response following an infected bite that is markedly different from that observed in naïve animals. In contrast to naïve mice, the parasites co-deposited with saliva into the bite site of exposed animals rapidly encounter an influx of proinflammatory leukocytes that persist for a prolonged period after transmission. Of significance, the proinflammatory response of exposed mice parallels findings in a study recently conducted in humans naturally bitten by *P. duboscqi* [49]. Biopsies taken at the site of a DTH response 48 hours after experimental bites were dominated by lymphocytes, macrophages and high levels IFN-γ indicative of a Th1 response. Importantly, a DTH response to bites was predominant in naturally exposed humans being observed in 75% of individuals aged 1–15 years [49]. The profound modulation of the feeding site in exposed individuals underscores the importance of vector-derived factors in understanding the pathogenesis and control of vector-borne diseases.

This report represents the first description of the kinetics and nature of the local cellular infiltrate in saliva-exposed mice that are protected from disease following vector-transmitted *L. major*.
